# Systemic Treatment Initiation in Classical and Endemic Kaposi’s Sarcoma: Risk Factors and Global Multi-State Modelling in a Monocentric Cohort Study

**DOI:** 10.3390/cancers13112519

**Published:** 2021-05-21

**Authors:** Lina Benajiba, Jérôme Lambert, Roberta La Selva, Delphine Cochereau, Barouyr Baroudjian, Jennifer Roux, Jérôme Le Goff, Cécile Pages, Maxime Battistella, Julie Delyon, Céleste Lebbé

**Affiliations:** 1 Université de Paris, AP-HP, Clinical Investigations Center, INSERM U944, Saint Louis Hospital, 75010 Paris, France; 2 Université de Paris, AP-HP, Biostatistics Department, Saint Louis Hospital, 75010 Paris, France; jerome.lambert@univ-paris-diderot.fr; 3 A.O.U. Città della Salute e della Scienza di Torino, 10126 Turin, Italy; robertals@libero.it; 4 Université de Paris, AP-HP, Dermatology Department, INSERM U976, Saint Louis Hospital, 75010 Paris, France; delphine_cochereau@hotmail.fr (D.C.); barouyr.baroudjian@aphp.fr (B.B.); Jennifer.roux@aphp.fr (J.R.); pageslaurent.cecile@iuct-oncopole.fr (C.P.); julie.delyon@aphp.fr (J.D.); 5 Université de Paris, AP-HP, Microbiology Department, Saint Louis Hospital, 75010 Paris, France; jerome.le-goff@aphp.fr; 6 Université de Paris, AP-HP, Pathology Department, INSERM U976, Saint Louis Hospital, 75010 Paris, France; maxime.battistella@aphp.fr

**Keywords:** classical and endemic Kaposi Sarcoma, systemic treatment, multi-state modelling, treatment free interval, chemotherapy, interferon

## Abstract

**Simple Summary:**

Over the past decades, clinical features and patients’ outcome of iatrogenic and HIV-related KS epidemiological subtypes have been widely described in large cohort series. Due to their lower incidence and the limited resources available in endemic KS countries, classical and endemic KS epidemiological studies remain scarce, thus increasing the challenge of such clinically heterogeneous chronic diseases’ management. In this large retrospective cohort study, six risk factors for treatment initiation were identified: time between first symptoms and diagnosis ≥1 year, endemic KS, total number of lesions ≥10, visceral or head/neck localization and edema. No response or treatment-free time difference was observed between the most frequently used therapeutic options: chemotherapy and interferon-alpha. Assessment for systemic treatment risk factors provides guidance for adequate follow-up and patients’ information on disease outcome. Absence of efficacy difference between systemic regimens allows treatment choice based on fitness.

**Abstract:**

Background: Although several studies described the clinical course of epidemic and post-transplant Kaposi’s Sarcoma (KS), the lack of large cohorts of classic/endemic KS, precluded such characterization. Methods: We used multi-state modelling in a retrospective monocentric study to evaluate global disease evolution and identify risk factors for systemic treatment (ST) initiation. 160 classic/endemic KS patients consecutively diagnosed between 1990 and 2013 were included. Results: 41.2% of classic/endemic KS patients required ST. Cumulative incidence of ST after 2 years of follow-up was 28.4% [95% CI: 20.5; 35.5]. Multivariate analysis identified six risk factors for ST initiation: time between first symptoms and diagnosis ≥1 year, endemic KS, total number of lesions ≥10, visceral, head or neck localization and presence of edema. Type of ST, type of KS, age and time between diagnosis and ST were not associated with response. Mean treatment-free time during the first 5 years following ST was 44 months for interferon and 44.6 months for chemotherapy treated patients (Mean difference: −0.5 months [95% CI: −9.5; 4.9]). Conclusions: Our study reveals ST risk factors in classic/endemic KS and highlights the clinical aggressiveness of the endemic KS subtype. No efficacy difference was observed between standard of care treatments, enabling treatment choice based on patient’s fitness.

## 1. Introduction

KS is an HHV-8 related lympho-angioproliferative disease with 4 clinical settings: iatrogenic (immunosuppressive therapy related), epidemic (HIV immune-deficiency related), endemic, and classic. Endemic KS develops in Sub-Saharian Africans whereas classic KS typically affects middle to elderly Mediterranean men with a male to female ratio ranging from 2:1 to 5:1 and an estimated incidence of 1.58 per 100,000 inhabitants per year in Sardinia [1,2].

HHV-8, also called KS-associated herpes virus (KSHV) is a herpes virus mainly transmitted through prolonged or repeated saliva contact during mother to child or sexual interactions. After an initial replicative phase, HHV-8 enables immune system evasion and establishes latency in the KS tumors [3]. T cell immune suppression is a well-recognized risk factor for HIV and transplant associated KS. Similarly, defects in NK cell and HHV-8-specific CD8 cells activity have been reported in classic and endemic KS [4].

The four KS epidemiological subtypes account for a wide clinico-pathological disease spectrum with some patients experiencing an indolent form of the disease while others present an aggressive disseminated pattern. Clinically, KS manifests mainly as purple-blue pigmented macules, plaques or nodules in the skin. More rarely, KS can also involve mucosa, lymph nodes or visceral organs such as gastro-intestinal tractus, lungs, bones and liver. Classic and endemic KS are typically indolent and mainly presents as limb lesions, with less than 10% mucosal, visceral or lymph node involvement [5]. HIV and iatrogenic KS are usually more disseminated in the skin and frequently involve mucosa, lymph nodes and visceral organs [6,7].

KS treatment remained mainly unchanged over the past 30 years. Management is based on: number and localization of lesions, presence of symptomatic lesions, disease progression and patient’s fitness [8]. Patients with asymptomatic lesions are usually offered careful observation. Those presenting with symptomatic superficial or isolated skin lesions are treated locally, while more extensive, disseminated or visceral locations are treated systemically. Although such systemic strategies, based on interferon or chemotherapy, usually result in a 50 to 80% overall response rate, they are not curative and their efficacy is only transient [8].

The low incidence of classic/endemic KS, combined with the limited resources available in the endemic KS countries has precluded conducting large therapeutic studies in these KS subtypes. Non-HIV related KS subtypes management is thus mainly based on small retrospective clinical series, physicians experience and consensus based multidisciplinary guidelines [8,9]. No large real-life data are available to our knowledge to help clinicians’ choice on systemic therapy decisions.

Aiming to improve endemic/classic KS management, we conducted a large monocentric retrospective study, in order to describe the disease’s clinical course, identify risk factors for systemic treatment initiation and evaluate the rate and duration of response to systemic therapy, in a real-life cohort of classic/endemic KS patients. To our knowledge, this is the first study to report risk factors for systemic treatment initiation and to use multi-state modelling to evaluate global disease evolution in this rare KS subtypes. Our study should inform clinicians on the clinical course of classic/endemic KS and provide guidance in therapeutic management. Overall our findings should improve patients’ quality of life and experience with this rare and chronic KS subtypes.

## 2. Materials and Methods

### 2.1. Study Design and Study Population

We performed a retrospective monocentric study, including all patients consecutively diagnosed with classic or endemic KS (*n* = 160) within one French dermato-oncology center between January 1990 and December 2013. Patients diagnosed prior to January 1990 have been excluded from the study to avoid discrepancies in the treatment modality due to absence of use of interferon-alpha as a first-line treatment in KS at the time. All histologically proven KS without any context of HIV or other causes of iatrogenic immunosuppression were enrolled in this study and analyzed. KS was then sub-classified into endemic or classical subtypes according to Lebbé et al. [8]. Patients work up at diagnosis included an exhaustive clinical examination, chest radiography, abdomen ultrasound as well as white blood cell count, protein electrophoresis and check for the negativity of HIV. Additional work up was performed on an individual basis depending on patient symptoms.

The study was performed in accordance with the ethical guidelines of the Declaration of Helsinki. Patients provided informed consent. A unique anonymized database was established and homed in a secured system, meeting the security standards required by the protection of personal data law promulgated on 20 June 2018 in France. A diagnosis of Compliance and Security Research was carried out and approved by the data protection reference department of Saint-Louis hospital.

### 2.2. Endpoints

Clinical and biological features at time of KS diagnosis, local and systemic therapies with respective responses and toxicity were collected using a specific case report form (CRF), based on patient’s clinical records including both medical charts and electronic medical records. Clinical response was evaluated clinically and/or radiologically by the physician in charge of the patient. Complete Response (CR) was defined as complete clearance of all KS lesions, Partial Response (PR) was defined as a decrease in lesions area >50%, while Progressive Disease corresponded to patients with >25% lesions increase. All remaining patients were considered in Stable Disease (SD). The best overall response corresponds to the best response at any time during the assessment period. Local treatments were grouped as follows: surgery, local chemotherapy (imiquimod, fluorouracil or alitretinoin), radiotherapy or others (photodynamic therapy, cryotherapy, laser therapy, compression). Systemic treatments were grouped as follows: low dose interferon, chemotherapy (taxanes or anthracyclines-based regimens) or others (everolimus, thalidomide, lenalidomide, sunitinib, imatinib, ribavirine, ganciclovir or lopinavir).

### 2.3. Statistical Analysis

Quantitative variables are reported as median and interquartile range, while qualitative variables are reported as number and percentage.

Time to systemic treatment initiation was estimated using initial KS diagnostic date as origin. Since only 2 patients died without receiving any systemic treatment, this competing risk was not taken into account and these patients were censored at their time of death when estimating the cumulative incidence of systemic treatment initiation. We used a parametric modelling of the cumulative incidence of treatment initiation, using a Weibull distribution, to examine whether the risk of treatment initiation was constant across time. Association between baseline characteristics and systemic treatment initiation was estimated using a Cox proportional hazard model. To identify independent predictors of systemic treatment initiation, we then constructed a multivariate model, including all variables significantly associated in the univariate analysis and with less than 20% of missing data, and using a stepwise AIC-based variable selection.

We compared characteristics at time of systemic treatment initiation between responding patients (CR or PR as best overall response) and non-responding patients (SD or PD as best overall response) to first line of systemic treatment using Wilcoxon test or Fisher’s exact test.

To assess the global evolution of the disease, we modelled the whole course of treatment using multi-state modelling. In this setting, a patient can go from the state “treated” to “non-treated” and vice versa several times during his follow-up, and ultimately go to the absorbing state of death. Transition probabilities between treated and non-treated were calculated using the Nelson Aalen estimator, and we calculated the mean time spent treatment free, truncated to some fixed limit tau. This mean time spent treatment-free was calculated starting from the beginning of first systemic treatment until either 1 year or 5 years following this treatment initiation. This treatment-free time was compared between groups according to KS subtype or first line of systemic treatment. The mean difference truncated at 1 or 5 years was calculated along with its bootstrapped 95% confidence interval.

All statistical analyses were performed using R software (version 3.6.1).

## 3. Results

This section may be divided by subheadings. It should provide a concise and precise description of the experimental results, their interpretation, as well as the experimental conclusions that can be drawn.

### 3.1. Demographic Characteristics

A total of 160 patients with histologically proven KS were included in the study. Patient’s characteristics are described in Table 1; 131 patients (81.9%) had classic KS and 29 patients had endemic KS. Median age was 62.6 years (IQR: 54.5; 72.4) and 87.5% of patients were males. Lower limbs were the most commonly involved region (91.2%). The majority of patients (55.0%) had less than 10 lesions, and 10% had an extensive skin disease with over 100 KS lesions. Almost half of the patients (46.3%) presented with lymphedema, and 21.2% patients had symptomatic painful lesions.

HHV-8 viral quantification was available for 118 patients at diagnosis. Among them, 22.9% patients had a positive peripheral blood viral load with a median HHV-8 viral load of 3.22 log (IQR: 2.50; 3.59). Lymphopenia (total lymphocytes count <1500/mm^3^) was present in 48.7% patients, and CD4 median count was 701/mm^3^ (IQR: 507; 913). Serum LDH level was above upper normal limit in 18.6% of patients.

### 3.2. Risk Factors for Systemic Treatment Initiation

With a median follow-up of 4.8 years, 13.8% of patients did not require any treatment while 45.0% and 41.2% of them required local or systemic treatments respectively. Local treatment consisted in surgery (36.1%), local chemotherapy (29.5%), radiotherapy (26.2%) or other (8.2%). Systemic treatments included low dose interferon (50.0%), chemotherapy (taxanes or anthracyclines-based regimens) (45.5%) or other therapies (4.5%). Among the 66 patients who required systemic treatment, 53% received more than one line of treatment.

**Figure 1:**
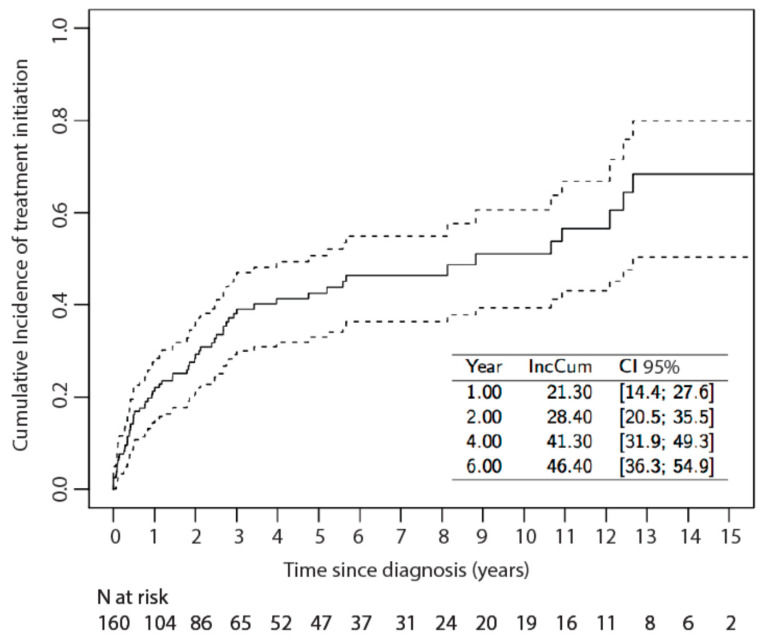
Cumulative Incidence of systemic treatment initiation in classic/endemic KS patients. Dashed lines correspond to the 95% Confidence Interval (CI). The inset table reports the cumulative incidences (IncCum) at 1, 2, 4 and 6 years after KS diagnosis, with their 95% CI.

Cumulative incidence of systemic treatment initiation after 2 years of follow-up was 28.4% (95% CI: 20.5; 35.5), and median time from KS diagnosis to systemic treatment initiation was 8.8 years (95% CI: 4.7; 12.7) (Figure 1). Parametric modelling of the cumulative incidence showed that instantaneous risk of systemic treatment initiation decreases over time.

Among baseline variables, endemic KS subtype, total number of cutaneous lesions, disease localization, painful lesions, lymphedema, and elevated serum LDH levels were significantly associated with systemic treatment initiation in univariate analysis (Table 2).

**Figure 2:**
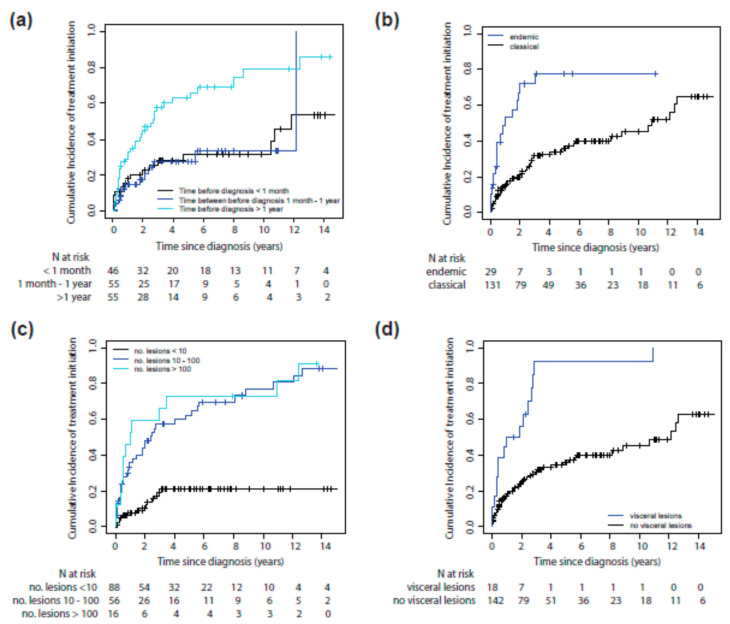

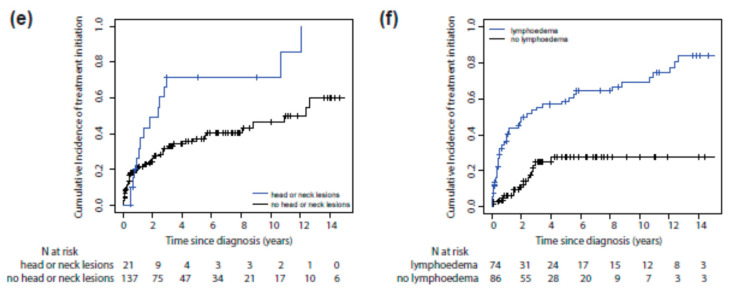
Cumulative Incidence of systemic treatment initiation in classic/endemic KS patients according to: time between first symptoms and diagnosis (**a**), KS subtype (endemic vs. classic) (**b**), total number of lesions (**c**), visceral localization (**d**), head/neck localization (**e**), or presence of lymphedema (**f**).

Six risk factors for systemic treatment initiation were identified with multivariate analysis: endemic versus classic KS (HR: 3.29 [95% CI: 1.71; 6.36]), total number of lesions higher than 10 {HR: 3.64 (95% CI: 1.81; 7.35)}, visceral localization (HR: 2.11 [95% CI: 1.06; 4.18]), head or neck localization (HR: 2.21 [95% CI: 1.15; 4.25]), presence of edema (HR: 2.18 [95% CI: 1.18;4.04]) and a time between first symptoms and diagnosis longer than 1 year (HR: 2.70 [95% CI: 1.41; 5.17] for more than 1 year) (Figure 2a–f and Table 2).

### 3.3. Therapeutic Response to KS Systemic Treatment

**Figure 3:**
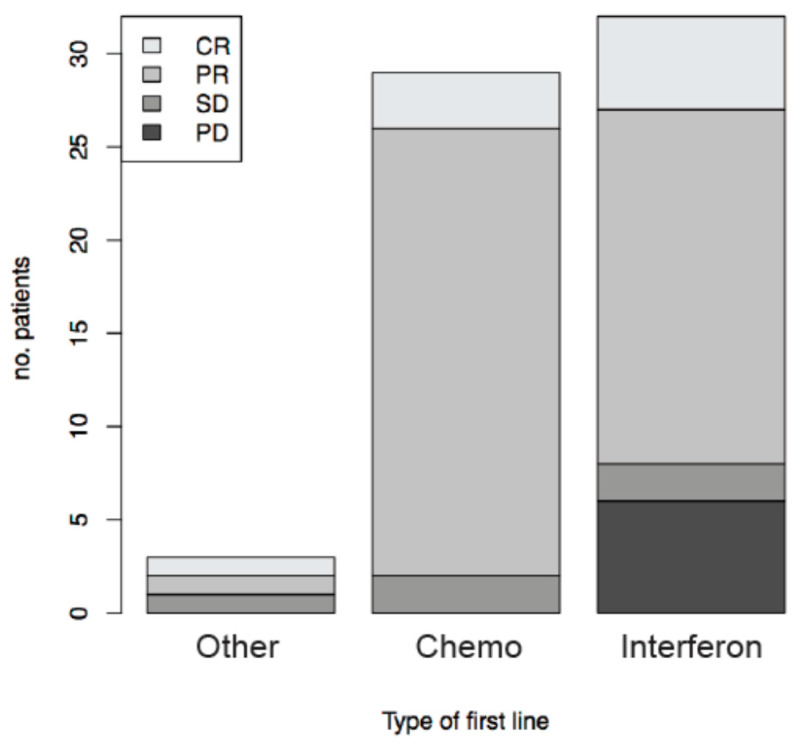
Best overall response after first line of systemic treatment in classic/endemic KS patients, according to type of therapy. Chemo: chemotherapy. CR: Complete Response. PR: Partial Response. SD: Stable Disease. PD: Progressive Disease.

Among the 66 patients who received at least one line of systemic treatment for KS, best overall response (BOR) after the first line of systemic treatment was available for 64 patients. 14% of patients had a complete response (CR) while partial response (PR) was observed in 69%, stable disease (SD) in 8% and progressive disease (PD) in 9% of patients. BOR after first line of systemic treatment, according to type of therapy (interferon, chemotherapy or other regimens) is presented in Figure 3. 93.1% and 75.0% of patients treated respectively with chemotherapy or low-dose interferon achieved an objective response (CR or PR).

Type of first line therapy (low dose interferon, chemotherapy or other), type of KS (endemic or classic), age at therapy initiation and time between diagnosis and systemic treatment initiation were not associated with BOR (Table A1).

### 3.4. Treatment Free Time after KS Systemic Treatment

**Figure A1:**
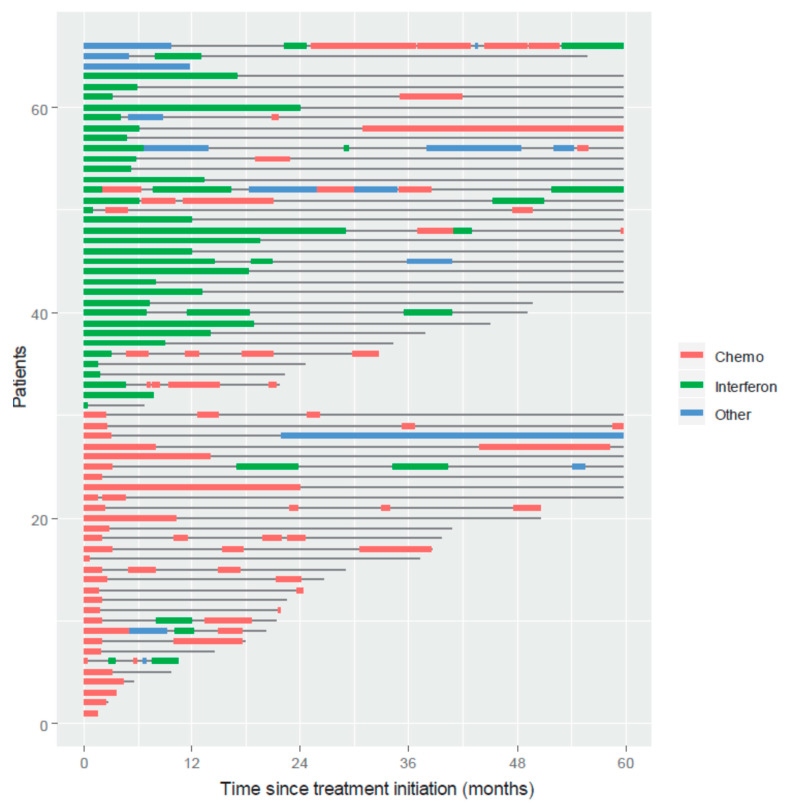
Lollipop plot representing treatment course of the 66 classic/endemic KS patients who received systemic treatment. Each line represents a single patient. Treatment course is represented during the first five years of follow-up. Treatment free periods are represented by a grey line while treatment intervals correspond to green, red or blue boxes when patients are treated with interferon, chemotherapy or other regimens respectively.

Given the chronic evolution of KS and the impact of systemic treatment on the quality of life, we explored the treatment course of the 66 classic/endemic KS patients who received systemic treatment (Figure A1).

**Figure 4:**
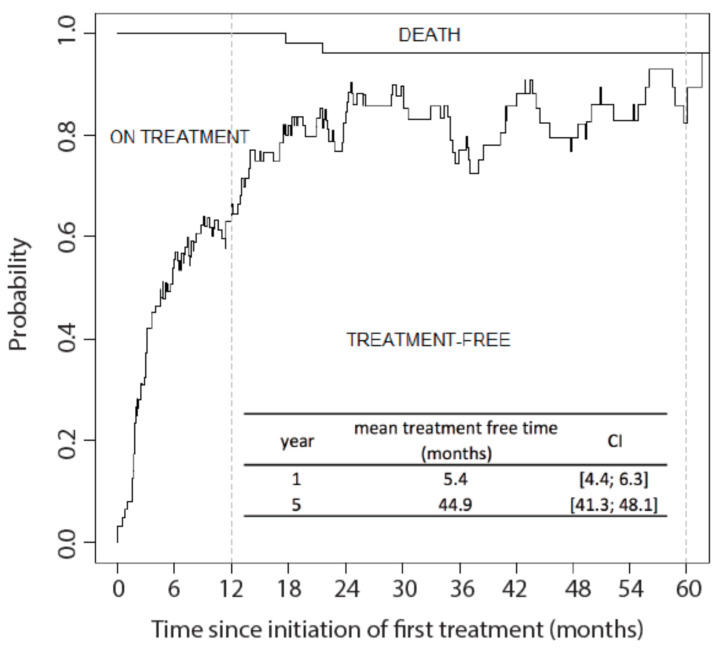
Treatment free time after systemic treatment initiation in classic/endemic KS. This figure was generated using state occupation probabilities: the area under the curve represents the mean time spent alive, on treatment and alive treatment-free during the first 5 years following the initiation of first systemic treatment. The inset table reports the mean times spent treatment free during the first year and the first 5 years following the initiation of first systemic treatment in the whole cohort, with their 95% CI. Dotted lines correspond to 1 and 5 years after first treatment.

Multi-state modelling was used to study the whole course of treatment. The mean time spent treatment free was calculated starting from the beginning of first systemic treatment until either 1 year or 5 years following treatment initiation. The mean cumulative treatment-free time during the first year and the first 5 years following systemic treatment initiation was 5.4 months [95% CI: 4.4; 6.3] and 44.9 months [95% CI: 41.3; 48.1] respectively (Figure 4).

**Figure 5:**
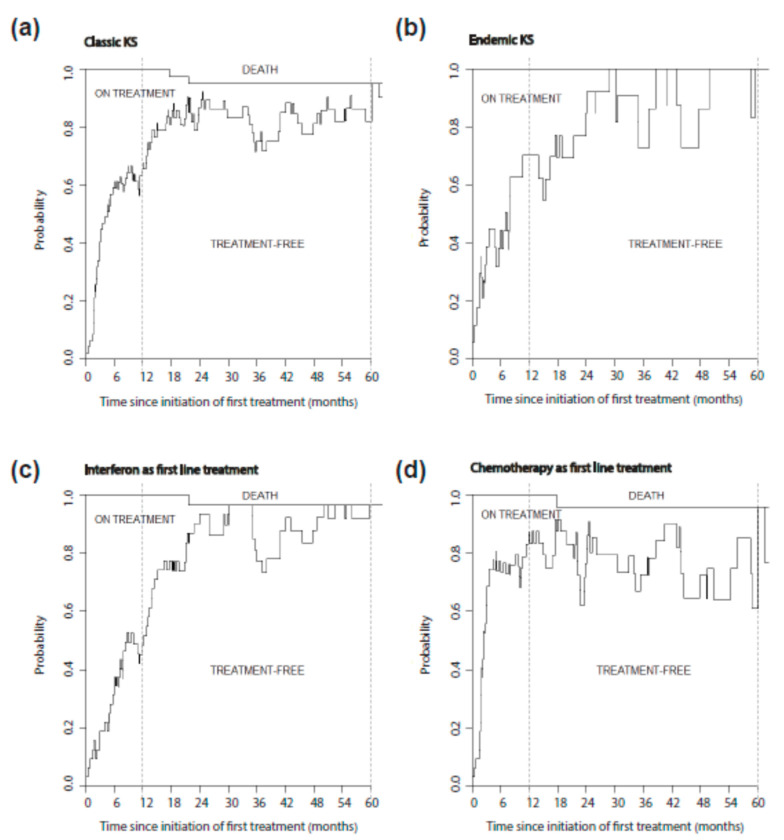
Treatment free time after systemic treatment initiation in classic/endemic KS patients stratified according to KS subtype: classic (**a**) or endemic (**b**), or according to type of first line treatment: interferon (**c**) or chemotherapy (**d**). This figure was generated using state occupation probabilities: the area under the curve represents the mean time spent alive, on treatment and alive treatment-free during the first 5 years following the initiation of first systemic treatment. Dotted lines correspond to 1 and 5 years after first treatment initiation.

The mean treatment-free time during the first and the 5 first years post-treatment initiation was not significantly different between classic (5.6 and 45.3 months at 1 and 5 years respectively) and endemic KS (4.8 and 43.5 months at 1 and 5 years respectively) (mean difference at 1 year: 0.8 months [95% CI: −4.9; 9.1], mean difference at 5 years: 1.7 months [95% CI: −1.4; 3.0]) (Figure 5a,b).

During the first-year post-treatment initiation, the mean treatment-free time was higher in chemotherapy-treated patients (7.3 months) compared to interferon-treated patients (3.5 months) (mean difference: −3.8 months [95% CI: −6.0; −2.7]). However, this difference was no longer observed at 5 years post-treatment initiation (chemotherapy: 44.6 months, interferon: 44.0 months, mean difference: −0.5 months [95% CI: −9.5;4.9]), suggesting that the early difference observed is mainly related to a difference in treatment regimens length, rather than a real impact of either regimen on the disease course (Figure 5c,d).

## 4. Discussion

Our study provides an overview of clinical characteristics and therapeutic outcome in a large real-life endemic/classic KS cohort. Although classic/endemic KS are thought to have an indolent disease course, they can become symptomatic and require the use of systemic therapy [8]. In our study, 41.2% patients required the use of systemic therapy. This proportion reflects the recruitment of our specialized outpatients’ clinic and may overestimate the incidence of systemic treatment requirement in a non-hospital dermatology consult.

Defining an adequate follow-up frequency adapted to the clinical course of endemic/classic KS, remain challenging for physicians taking care of KS patients [8].

There is no universally accepted staging classification for endemic/classic KS. Three staging systems have been proposed but deserve further validation and are not commonly used in the real-life. They take into account KS lesions localization and skin lesions number, extension and evolution [10,11,12]. Our results pinpoint a subpopulation of KS patients with high risk of systemic treatment requirement. Endemic KS, presence of more than 10 lesions, visceral or head/neck localization, or presence of lymphedema define objective criteria for systemic treatment initiation. These results should allow clinicians to better adjust follow-up schedules depending on susceptibility of systemic treatment need at diagnosis. Additionally, this will help physicians better inform patients on the clinical course of their disease.

Interestingly, our study shows that time from diagnosis to systemic treatment initiation does not influence response to treatment. This result allows physicians to adapt treatment initiation to patient’s quality of life requirements, as KS is a chronic disease with no curative therapeutic options available to date, the main objective of systemic therapy remaining an improved quality of life [8]. Physical or psychological repercussions of KS lesions can indeed be responsible of a considerably reduced quality of life due to esthetic considerations, pain, edema or visceral symptoms, mainly gastro-intestinal and pulmonary [8]. Moreover, our data suggest that response to first-line systemic treatment does not depend on patients’ age at treatment initiation. Thus, age should not preclude clinicians from treatment initiation if patients are considered fit to receive the proposed treatment. Systemic treatment initiation should thus be adapted to each patient, balancing its initial risk of systemic treatment requirement, patient’s fitness and presence of quality of life impacting symptoms.

KS systemic therapy mainly relies on chemotherapeutic agents such as liposomal doxorubicin [13,14,15,16] or taxanes [17,18], and immune-modulating therapies such as low-dose interferon alpha and its pegylated derivatives, mainly used for younger patients with classic KS [19,20,21]. Our study did not reveal any significant difference in overall response rates, nor in treatment free time, between the two more frequently used regimens to treat endemic/classic KS: low dose interferon and taxanes or anthracyclines-based chemotherapy. Response rates in our study were in line with previous smaller scale studies [9]. Although both treatments are efficient, they are associated with significant relapse rates and are usually not curative. Aiming to highlight the therapeutic option offering the best quality of life, we further explored response to these two systemic treatment options in terms of response duration, through treatment free time evaluation. This is to our knowledge the first study comparing treatment-free time between both therapeutic options. Although treatment-free time was higher in the chemotherapy treated population during the first year, interferon- and chemotherapy-treated patients had similar treatment-free intervals on a long-term perspective. These results only reflect the longer duration of interferon regimens compared to short chemotherapeutic regimens and suggest that neither interferon nor chemotherapy-based regimens offer a longer duration of response. Further studies focusing on systemic treatment toxicities in the endemic/classic KS population should further inform on treatment choice depending on patient’s fitness. Treatment choice should thus be adapted to patient’s fitness to avoid specific toxicities and improve patient’s quality of life.

Endemic KS develops in younger patients and tends to be difficult to treat and have poor clinical outcome with high lymphedema rates [22,23]. To our knowledge, this is the first report of a large series of non-HIV related and non-iatrogenic KS, highlighting the endemic subtype clinical aggressiveness. Although patients harboring the endemic subtype had a higher systemic treatment requirement rate in our study, they respond similarly to first-line therapy once initiated, both in terms of response rates and duration.

Finally, as KS therapy is now entering an exciting immunotherapy avenue, our data offers a baseline for clinical characteristics and outcome of endemic/classic KS in the pre-immunotherapy era. Indeed, KS tumors strongly express the T cell inhibitory molecules PD1 and PDL1 [24] and several promising results have recently emerged from pilot reports testing immune checkpoint inhibitors (ipilimumab and/or nivolumab), mainly in HIV-associated Kaposi Sarcoma (NCT02408861) [25,26,27]. Further anti-PD1/PDL1 clinical studies should inform on the efficacy of these agents compared to the standard chemotherapy and low-dose interferon regimens, especially in the long term.

Limitations of our study include its monocentric setting within a specialized outpatient’s clinic. Proportion of patients requiring a systemic treatment may therefore be overestimated, and KS management heterogeneity across different centers needs to be taken into account in our study results interpretation. The wide period of patient’s inclusion ranging from 1990 to 2013 also represents a limitation as this might be responsible for some degree of heterogeneity in terms of first-line treatment modalities.

## 5. Conclusions

In this study, we report the clinical characteristics and outcome of a large monocentric cohort of endemic and classic KS. The endemic subtype, total number of lesions, visceral or head/neck localization, presence of edema at diagnosis and a higher time between first symptoms and diagnosis were independent risk factors for systemic treatment initiation. No response difference was observed between the standard of care treatments, chemotherapy and interferon, thus promoting the guidance of therapeutic choice following patient’s comorbidities.

## Figures and Tables

**Table 1:** Patients’ characteristics at KS diagnosis. UNL: Upper Normal Limit. IQR: Inter-Quartile Range. LDH: Lactate Dehydrogenase.

Patients Characteristics (Total)	*n* = 160
Age at diagnosis (years), median (IQR)	62.6 (54.5; 72.4)
Gender	
female	20 (12.5%)
male	140 (87.5%)
Subtype of Kaposi Sarcoma	
classic	131 (81.9%)
endemic	29 (18.1%)
Number of lesions	
0–10	88 (55%)
10–100	56 (35%)
>100	16 (10%)
Disease localisation	
lower limbs	146 (91.2%)
upper limbs	61 (38.1%)
trunk	32 (20.1%)
head or neck	21 (13.3%)
mucosa	12 (7.5%)
visceral	18 (11.2%)
Painful lesions	
yes	33 (21.2%)
no	123 (78.8%)
Lymphedema	
yes	74 (46.3%)
no	86 (53.7%)
Serum LDH (*n* = 97)	
<UNL	79 (81.4%)
>UNL	18 (18.6%)
HHV8 PCR (*n* = 118)	
positive	27 (22.9%)
negative	91 (77.1%)
viral load (log), median (range)	3.22 (2.50; 3.59)
Lymphocytes count (*n* = 117)	
>1500/mm^3^	60 (51.3%)
<1500/mm^3^	57 (48.7%)
CD4 count (nb/mm^3^), median (IQR) (*n* = 76)	701 (507; 913)
Treatment	
observation	22 (13.8%)
local	72 (45%)
systemic	66 (41.2%)

**Table 2:** Univariate and Multivariate COX model identifying risk factors for systemic treatment initiation in patients with classic/endemic KS. HR: Hazard Ratio. CI: Confidence Interval.

		Univariate Analysis			Multivariate Analysis	
	HR	CI 95%	*p*-Value	HR	CI 95%	*p*-Value
Age at diagnosis (years)	1	(0.98–1.02)	8.62 × 10^−1^			
Time from first symptoms to diagnosis (months)						
<1 m	1		4.98 × 10−^4^	1		6.82 × 10^−3^
1–12 m	0.98	(0.47–2.05)		2.19	(0.98; 4.91)	
>12 m	2.61	(1.44–4.72)		2.7	(1.41; 5.17)	
Subtype of Kaposi Sarcoma						
classic	1		4.56 × 10^−5^	1	.	7.59 × 10^−4^
endemic	3.61	(2.07–6.3)		3.29	(1.71; 6.36)	
Number of lesions						
0–10	1		2.26 × 10^−9^	1		1.27 × 10^−4^
10–100	5.2	(2.82–9.6)		3.64	(1.81; 7.35)	
>100	6.17	(2.89–13.18)		4.56	(1.98; 10.54)	
Disease localization						
lower limbs no	1		4.76 × 10^−3^			
yes	7.25	(1.01–52.24)				
upper limbs no	1		2.82 × 10^−3^			
yes	2.1	(1.29–3.41)				
trunk no	1		1.99 × 10^−2^			
yes	1.93	(1.14–3.29)				
head or neck no	1		1.12 × 10^−2^	1		2.34 × 10^−2^
yes	2.26	(1.26–4.06)		2.21	(1.15; 4.25)	
mucosa no	1		8.50 × 10^−2^			
yes	1.96	(0.97–3.97)				
visceral no	1		2.32 × 10^−5^	1	.	3.98 × 10^−2^
yes	4.1	(2.3–7.31)		2.11	(1.06; 4.18)	
Painful lesions						
no	1		1.17 × 10^−3^			
yes	2.41	(1.45–4.02)				
Lymphedema						
no	1		2.52 × 10^−7^	1		5.62 × 10^−3^
yes	3.89	(2.23–6.79)		2.3	(1.25; 4.26)	
Serum LDH						
<ULN	1		3.42 × 10^−3^			
>ULN	2.76	(1.47–5.17)				
Lymphocytes count						
>1500/mm^3^	1		2.99 × 10^−1^			
<1500/mm^3^	0.75	(0.43–1.3)				

## Data Availability

All data supporting reported results is included in the main manuscript.

## Appendix A

**Table A1:** Factors associated to first systemic treatment line best overall response. CR: Complete Response. PR: Partial Response. SD: Stable Disease. PD: Progressive Disease.

	CR or PR (*n* = 53)	SD or PD (*n* = 11)	*p*-Value
Age at treatment (years), median (IQR)	64.5 (57.7; 73.4)	58.3 (51.8; 74.1)	0.48
Time from diagnosis to systemic treatment (days), median (IQR)	428 (142; 1026)	306 (86; 830)	0.54
Subtype of Kaposi Sarcoma			1
classic	39 (74%)	8 (73%)	
endemic	14 (26%)	3 (27%)	
Type of systemic treatment			0.11
chemotherapy	27 (51%)	2 (18%)	
interferon	24 (45%)	8 (73%)	
other	2 (4%)	1 (9%)	
